# Analysis of preoperative risk factors for clinical decision-making in female upper urinary tract stones: a retrospective cohort study

**DOI:** 10.1186/s12894-026-02152-y

**Published:** 2026-04-23

**Authors:** Zimei Mo, Beixun Xu, Yang Chen, Puzhao Liang, Yijun Fu, Xin Gao, Yongtong Ruan

**Affiliations:** Yangjiang Hospital of Traditional Chinese Medicine, Affiliated with Guangzhou University of Chinese Medicine, Yangjiang, China

**Keywords:** Female patients, Upper urinary tract calculi, Risk factors, Clinical decision-making, Preoperative evaluation

## Abstract

**Objective:**

To identify independent risk factors that can be used for the preoperative prediction of treatment complexity in female patients with upper urinary tract stones, thereby providing a basis for clinical decision-making.

**Methods:**

This single-center retrospective cohort study consecutively enrolled 219 female patients with upper urinary tract stones admitted to the Department of Urology between January 2024 and May 2025. Based on the occurrence of treatment complexity (including: ① preoperative ureteral stent placement due to infection or obstruction; ② ≥3 stone-related readmissions within 12 months; ③ hospital stay > 6 days), patients were categorized into a complex course group (*n* = 107) and a control group (*n* = 112). Preoperative clinical data were collected, and univariate and multivariate logistic regression analyses were performed to identify independent risk factors. Statistical analysis was performed using SPSS version 25.0. An exploratory preoperative risk stratification framework was proposed based on the results of multivariate analysis.

**Results:**

Univariate analysis revealed significant differences between the two groups in age (*P* = 0.008), stone location (*P* < 0.001), stone area (*P* = 0.039), presence of bacteria/fungi (*P* < 0.001), acute renal insufficiency (*P* < 0.001), high-sensitivity C-reactive protein (hs-CRP) level (*P* = 0.001), procalcitonin (PCT) level (*P* < 0.001), body mass index (BMI), history of previous urological surgery, ureteral stenosis, bilateral stones, comorbid diabetes, hypertension, fatty liver, white blood cell count, absolute neutrophil count, and neutrophil percentage. Multivariate analysis identified four independent risk factors: [1] Stone location (using ureteral stones as reference, renal stones: OR = 6.67, 95% CI: 2.67–16.68, renal-ureteral stones: OR = 3.37, 95% CI: 1.29–8.80) [2]. Bacterial/fungal infection (OR = 3.30, 95% CI: 1.42–7.62) [3]. hs-CRP level (OR = 1.01, 95% CI: 1.00-1.02) [4]. PCT level (OR = 1.12, 95% CI: 1.01–1.23). Based on these findings, an exploratory preoperative risk stratification framework was proposed. In this framework, bacterial/fungal infection and renal stone location were categorized as high-risk factors, while hs-CRP ≥ 7.3 mg/L and PCT ≥ 0.65 ng/mL were categorized as intermediate-risk factors, pending external validation.

**Conclusion:**

Bacterial/fungal infection, stone location (renal stones), elevated hs-CRP, and elevated PCT are effective preoperative predictors of treatment complexity in female patients with upper urinary tract stones. The proposed exploratory risk stratification framework may aid in the identification of high-risk patients and offers preliminary evidence to support individualized surgical strategies and perioperative management plans, though prospective validation is required before clinical implementation.

## Introduction

Urolithiasis is a highly prevalent urological disorder worldwide, with its occurrence rate demonstrating a significant upward trend over the past three decades. The lifetime prevalence of kidney stones has reached 14% globally, and the incidence and prevalence rates have shown a continuous increase in almost all countries [1]. This increase is closely associated with lifestyle changes (e.g., high-protein, high-salt diets, rising obesity rates) and advancements in diagnostic techniques [2]. Notably, the gender disparity pattern in stone disease incidence is being reconfigured. Traditionally, the prevalence was significantly higher in males than in females (male-to-female ratio 3:1); however, recent data indicate this ratio has decreased to 1.3:1, suggesting a marked increase in female patients [3]. In the therapeutic domain, the management of upper urinary tract calculi has comprehensively shifted from open surgery to minimally invasive techniques, establishing a stepped treatment plan centered on Percutaneous Nephrolithotomy (PCNL) and Retrograde Intrarenal Surgery (RIRS) [4]. Nevertheless, technological progress has not fundamentally alleviated the disease burden. Upper urinary tract calculi still result in over one million emergency department visits annually, and the risk of post-operative infection-related complications remains higher in female patients compared to males [5]. However, it is important to clarify that female sex serves primarily as a risk modifier rather than an independent determinant of infectious complications. The absolute risk is strongly influenced by modifiable factors, including preoperative urine culture status, the presence of infection stones, and operative parameters such as surgical duration and intrarenal pressure management. The increased susceptibility in female patients may stem from anatomical factors, hormonal influences on stone composition and urinary tract defense mechanisms, and a higher prevalence of infection stones [6, 7]. Consequently, a subset of female patients experiences complex clinical courses, characterized by events such as preoperative stent placement due to uncontrolled infection, multiple readmissions, or prolonged hospital stays. These events represent significant deviations from the ideal treatment pathway and consume healthcare resources. Female patients with upper urinary tract calculi face unique clinical challenges. Given the increasing proportion of female patients and their distinct susceptibility to infection-related complications, we designed this study as a clinically relevant subgroup analysis focusing on female patients with upper urinary tract calculi. The objective was to identify preoperative risk factors for treatment complexity within this specific population, rather than to establish sex-specific predictors. This approach allows for a more targeted assessment of the unique clinical challenges faced by female patients and provides evidence to support individualized clinical decision-making in this subgroup. However, research gaps persist in predicting perioperative risks specifically for this population. Traditional risk assessments often focus on postoperative complications, and tools capable of preoperatively identifying patients likely to experience complex treatment courses (e.g., requiring staged surgery due to severe infection, recurrent readmissions) are limited. Existing models (such as the S.T.O.N.E. or CROES scores) were not developed to address gender-specific risks, particularly those related to infection and inflammation [8]. Such treatment complexity not only affects patient recovery but also increases the healthcare burden. Therefore, exploring a preoperative risk assessment tool based on routine preoperative indicators is crucial for optimizing clinical decision-making, including the timing of surgery, application of prophylactic antibiotics, and patient counseling. This study aimed to systematically investigate preoperative risk factors for treatment complexity in female patients with upper urinary tract stones through a retrospective cohort analysis. An exploratory preoperative risk stratification framework was proposed to help identify high-risk patients and provide preliminary evidence to support individualized surgical strategies and perioperative management. Prospective external validation is required before this framework can be applied in clinical practice.

## Materials and methods

### Data source

This single-center retrospective cohort study was approved by the Institutional Ethics Committee (Approval No.: 202500617001), and the requirement for informed consent was waived. Female patients with upper urinary tract stones who underwent surgical procedures (including PCNL and RIRS) in the Department of Urology of our hospital between January 2024 and May 2025 were consecutively enrolled.

The inclusion criteria were as follows: ① all patients were female. ② age ≥ 18 years. ③ diagnosis of upper urinary tract stones confirmed by imaging examinations such as ultrasonography or CT, with concomitant surgical indications. ④ availability of complete clinical data and ⑤ patients scheduled for or having undergone minimally invasive surgery for upper urinary tract stones, or those who had previously undergone ureteral stent placement.

The exclusion criteria were as follows: ① patients with upper urinary tract stones who opted for conservative management or extracorporeal shock wave lithotripsy. ② patients who underwent only ureteral stent removal at our hospital. ③ patients with contraindications to surgery, such as severe cardiopulmonary insufficiency and ④ cases lost to follow-up due to refusal of surgery during hospitalization.

### Outcome definition

The primary outcome was a composite endpoint termed “treatment complexity,” which encompasses three domains reflecting different aspects of the clinical course: preoperative decision-making and procedural utilization, postoperative healthcare resource utilization, and recovery trajectory. Specifically, the composite outcome was defined as the presence of any of the following criteria:

- ① Preoperative ureteral stent placement, defined as stent insertion prior to definitive stone surgery due to uncontrolled infection (e.g., refractory urinary tract infection or urosepsis) or severe pain causing obstruction, indicating a need for staged surgical management.
- ② Frequent stone-related readmissions, defined as three or more hospital readmissions within 12 months after the index surgery, attributed to stone-related events such as residual stones, urinary tract infection, or obstruction.
- ③ Prolonged hospital stay, defined as a total length of hospital stay exceeding 6 days, which corresponded to the median duration of hospitalization in this cohort and served as a proxy for a more complicated perioperative course.

These three components capture distinct but interrelated dimensions of treatment complexity: the need for additional preoperative interventions (stent placement), postoperative morbidity requiring repeated hospital care (frequent readmissions), and extended perioperative resource utilization (prolonged hospitalization).

### Data collection

A total of 219 female patients with upper urinary tract stones who underwent surgical treatment in the hospital’s Urology Department between January 2024 and May 2025 were retrospectively included. Variable selection encompassed three major pathological mechanisms: anatomical, metabolic, and infectious factors.

#### General factors

Patient age, stone location, stone area (mm², calculated as the maximum diameter multiplied by the maximum transverse distance), body mass index (BMI), history of previous urological surgery, concomitant ureteral stricture, and bilateral stones.

#### Comorbidities

Diabetes, hypertension, and fatty liver disease.

#### Biochemical indicators

Bacterial/fungal infection (defined as a positive urine or blood culture), acute renal insufficiency, high-sensitivity C-reactive protein (hs-CRP) level (positive if > 6 mg/L), white blood cell (WBC) count (positive if > 9.5 × 10⁹/L), absolute neutrophil count (positive if > 6.3 × 10⁹/L), neutrophil percentage (positive if > 75%), and procalcitonin (PCT) level (positive if > 0.5 ng/mL).

### Statistical analysis

Statistical analysis was performed using SPSS version 25.0. The normality of continuous variables was assessed using the Shapiro-Wilk test, with a significance level of α = 0.05. Continuous variables conforming to a normal distribution are presented as mean ± standard deviation and were compared using the independent t-test. Continuous variables not conforming to a normal distribution are expressed as median (first quartile, third quartile) [M (Q1, Q3)] and were compared using the Mann-Whitney U test. Categorical variables are presented as frequency (percentage) [n (%)] and were compared using the chi-square test. Categorical variables were numerically coded as follows: bacterial/fungal infection (0 = no, 1 = yes); stone location was dummy coded with ureteral stones as the reference category (coded as 3). Preliminary variable screening was conducted using univariate analysis, with *P* < 0.05 considered statistically significant. Variables with *P* < 0.05 in the univariate analysis were included in a binary logistic regression model using the Enter method. P-values, odds ratios (ORs), and 95% confidence intervals (CIs) were obtained to progressively identify independent risk factors. Multicollinearity was diagnosed using the variance inflation factor (VIF), with a threshold set at VIF < 5.

## Results

A total of 219 patients were enrolled, including 107 in the complex disease course group and 112 in the control group. Age conformed to a normal distribution (*P* = 0.524), whereas the remaining continuous variables—white blood cell (WBC) count, absolute neutrophil count, neutrophil percentage, body mass index (BMI), high-sensitivity C-reactive protein (hs-CRP), procalcitonin (PCT), and stone area—were all non-normally distributed (*P* < 0.05).

Among the 107 patients in the complex course group, the most frequently met component was prolonged hospital stay (> 6 days), observed in 74 patients (69.2%), followed by preoperative ureteral stent placement in 48 patients (44.9%), and frequent readmissions (≥ 3 episodes) in 22 patients (20.6%), as shown in Table 1.

**Table 1:** Distribution of the components of the composite outcome “treatment complexity”

Component	Complex Course Group (*n* = 107)	Control Group (*n* = 112)	Total (*N* = 219)
Preoperative ureteral stent placement	41 (38.3%)	(*n* = 112)	41 (38.3%)
≥ 3 stone-related readmissions within 12 months	17 (15.9%)	0 (0%)	17 (15.9%)
Hospital stay > 6 days	49 (45.8%)	0 (0%)	49 (45.8%)

### Univariate analysis of differences in clinical data between the complex course group and the control group

#### General related factors

A total of six risk factors were included. Among these, age (*P* = 0.008), stone location (*P* < 0.001), and stone area (*P* = 0.039) showed statistically significant associations with the complexity of treatment in female patients with upper urinary tract stones across the different groups. The mean age in the complex course group (57.30 ± 12.07 years) was significantly higher than that in the control group (53.11 ± 11.22 years), suggesting that advanced age may increase the risk of treatment complexity. Regarding the risk factor of stone location: the proportions of renal stones and combined renal/ureteral stones were significantly higher in the complex course group (30.84% vs. 7.14% and 19.63% vs. 8.04%, respectively), whereas the proportion of ureteral stones was significantly higher in the control group (49.53% vs. 84.82%). The stone area was larger in the complex course group [99.00 (35.00, 220.50) mm²] compared to the control group [63.00 (35.50, 120.00) mm²]. No statistically significant associations were found between BMI, history of previous urological surgery, concomitant ureteral stricture, or bilateral stones and the complexity of treatment in female patients with upper urinary tract stones, as shown in Table 2.

**Table 2:** Univariate analysis of clinical characteristics in female patients undergoing surgical treatment for upper urinary tract stones

	adverse outcome group (*n* = 107)	a control group (*n* = 112)	t/z/χ^2^	*P*
Age (years)	57.30 ± 12.07	53.11 ± 11.22	-2.66	0.008
BMI(kg/m^2^)	24.20 (21.90,27.30)	24.00 (21.90,25.50)	-0.64	0.519
Ureteral stenosis			1.37	0.242
No	81 (75.70%)	92 (82.14%)		
Yes	26 (24.30%)	20 (17.86%)		
Diabetes mellitus			3.71	0.054
No	88 (82.24%)	102 (91.07%)		
Yes	19 (17.76%)	10 (8.93%)		
Hypertension			2.52	0.113
No	74(69.16%)	88 (78.57%)		
Yes	33(30.84%)	24(21.43%)		
Bacterial/fungal infection			17.94	< 0.001
No	70 (65.42%)	100 (89.29%)		
Yes	37 (34.58%)	12 (10.71%)		
Hepatic steatosis			1.49	0.223
No	93 (86.92%)	103 (91.96%)		
Yes	14 (13.08%)	9 (8.04%)		
White blood cell count (WBC, ×10⁹/L)	7.99(6.21, 12.15)	8.86(6.28, 11.55)	-0.29	0.773
Absolute neutrophil count ( ×10⁹/L)	5.83(4.06, 10.09)	6.68(3.86, 9.52)	-0.15	0.880
	adverse outcome group (*n* = 107)	a control group (*n* = 112)	*t/z/χ* ^*2*^	*P*
Neutrophil percentage (%)	70.20(62.45, 85.15)	72.60(60.80, 82.80)	-0.40	0.691
History of urological surgery			1.80	0.179
No	81 (75.70%)	93 (83.04%)		
Yes	26 (24.30%)	19 (16.96%)		
Acute renal insufficiency			11.44	< 0.001
No	92 (85.98%)	110 (98.21%)		
Yes	15 (14.02%)	2 (1.79%)		
Bilateral calculi			2.74	0.098
No	59 (55.14%)	74 (66.07%)		
Yes	48 (44.86%)	38 (33.93%)		
Stone location			31.87	< 0.001
Renal stones	33 (30.84%)	8 (7.14%)		
Renal-ureteral stones	21(19.63%)	9 (8.04%)		
ureteral stones	53(49.53%)	95 (84.82%)		
High-sensitivity C-reactive protein (hs-CRP, mg/L)	7.27 (2.64, 53.49)	3.72 (1.83, 6.64)	-3.36	0.001
Procalcitonin (PCT, ng/mL)	0.10 (0.10, 0.65)	0.10 (0.10, 0.10)	-5.90	< 0.001
Stone area(mm²)	99.00(35.00,220.50)	63.00(35.50, 120.00)	-2.07	0.039

#### Factors related to underlying diseases

Three risk factors were included. The presence of diabetes, hypertension, or fatty liver showed no statistically significant association with the complexity of treatment in female patients with upper urinary tract stones, as shown in Table 2.

#### Factors related to biochemical indicators

A total of seven risk factors were analyzed. The proportion of bacterial/fungal infection was 34.58% in the complex course group, significantly higher than 10.71% in the control group (*P* < 0.001), suggesting that bacterial/fungal infection may be an important risk factor for treatment complexity in female patients with upper urinary tract stones. The incidence of acute renal insufficiency was significantly higher in the complex course group (14.02% vs. 1.79%, *P* < 0.001), indicating a strong association between acute kidney injury and treatment complexity. The median high-sensitivity C-reactive protein level was higher in the complex course group (7.27 vs. 3.72, *P* = 0.001). Although the median procalcitonin level was 0.10 in both groups, the difference was statistically significant (*P* < 0.001), with a higher frequency of elevated outliers (e.g., > 0.65) observed in the complex course group. Both findings suggest that elevated inflammatory markers are associated with treatment complexity. No statistically significant associations were found between white blood cell count, absolute neutrophil count, or neutrophil percentage and treatment complexity in female patients with upper urinary tract stones. See Table 2.

### Multivariate logistic regression analysis of independent risk factors for treatment complexity in female patients with upper urinary tract calculi

Variables with *P* < 0.05 in univariate analysis (age, bacterial/fungal infection, acute renal insufficiency, stone location, hs-CRP, PCT, and stone area) were includedinto a binary logistic regression model. The final model was statistically significant (*P* < 0.05). Stone location, bacterial/fungal infection, hs-CRP level, and procalcitonin level were identified as independent risk factors. Regarding stone location, using ureteral stones as the reference category, renal stones were associated with the highest risk (OR = 6.67, 95% CI: 2.67–16.68), followed by renal and ureteral stones (OR = 3.37, 95% CI: 1.29–8.80). Compared to patients without bacterial/fungal infection, those with infection had a significantly higher probability of treatment complexity (OR = 3.30, 95% CI: 1.42–7.62). For every 1 mg/L increase in hs-CRP level, the risk of treatment complexity increased by 1.3% (OR = 1.01, 95% CI: 1.00-1.02). Furthermore, the probability of treatment complexity increased with rising procalcitonin levels (OR = 1.12, 95% CI: 1.01–1.23). Age, acute renal insufficiency, and stone area were not statistically significant in the multivariate analysis (*P* > 0.05). The results are presented in Table 3.

**Table 3:** Multivariate Logistic Regression Analysis of Adverse Outcomes in Female Patients Undergoing Surgical Treatment for Upper Urinary Tract Calculi

	β	SE	Wald	*P*	OR	95% CI	
						Lower bound	Upper bound
Age	0.024	0.015	2.637	0.104	1.02	0.99	1.05
Bacterial/fungal infection	1.192	0.428	7.766	0.005	3.30	1.42	7.62
Stone location (reference: ureteral stones)			19.512	< 0.001			
Renal stones	1.898	0.468	16.483	< 0.001	6.67	2.67	16.68
Renal-ureteral stones	1.214	0.490	6.138	0.013	3.37	1.29	8.80
Acute renal insufficiency	3.295	3.295	3.295	0.081	4.28	0.84	21.91
High-sensitivity C-reactive protein (hs-CRP)	0.013	0.005	7.911	0.005	1.01	1.00	1.02
Procalcitonin (PCT)	0.109	0.050	4.862	0.027	1.12	1.01	1.23
Stone area	0.001	0.001	2.032	0.154	1.00	1.00	1.00
Constant	-2.679	0.835	10.292	0.001	0.07		

### Multicollinearity diagnosis

Multicollinearity was assessed using the variance inflation factor (VIF). All VIF values in the final model were < 3.0, indicating acceptable collinearity, as shown in Table 4.

**Table 4:** Collinearity Statistics

	tolerance	VIF
Age	0.879	1.137
Stone area	0.910	1.099
High-sensitivity C-reactive protein (hs-CRP)	0.889	1.124
Procalcitonin (PCT)	0.906	1.103
Bacterial/fungal infection	0.917	1.091
Acute renal insufficiency	0.909	1.100
Stone location	0.949	1.053

### Exploratory preoperative risk stratification framework

Based on the results of multivariate analysis, an exploratory risk stratification framework was constructed according to effect size (odds ratio) and clinical intervenability. External validation in an independent prospective cohort is required before this framework can be considered for clinical application.

High-risk factors:

1. Stone location (renal stones: OR = 6.67, 95% CI: 2.67–16.68).
2. Bacterial/fungal infection (OR = 3.30, 95% CI: 1.42–7.62).

Intermediate-risk factors (defined using data-driven cutoffs derived from the current cohort):

1. Procalcitonin (PCT) ≥ 0.65 ng/mL (the 75th percentile in the complex course group).
2. High-sensitivity C-reactive protein (hs-CRP) ≥ 7.3 mg/L (the median value in the complex course group).

The selection of these cutoffs was based on the distribution of biomarker levels within the current cohort and is therefore exploratory in nature. Their generalizability to other populations requires validation in independent external cohorts.

## Discussion

This study is the first to propose an exploratory preoperative risk stratification framework for treatment complexity in female patients with upper urinary tract calculi. This is motivated by the narrowing gender gap in the epidemiology of stone disease and the particular susceptibility of female patients to infection-related complications, which stems from factors such as anatomical structure, hormonal influences, and a higher prevalence of infection stones. Conducting subgroup analyses for this population can provide clinical insights complementary to existing risk models (e.g., S.T.O.N.E. or CROES scores), as these models were not specifically designed for gender-specific or infection-driven risk profiles [3, 6–8]. Our findings identified stone location (renal stones), preoperative bacterial/fungal infection, and elevated inflammatory markers (hs-CRP, PCT) as strong independent predictors of treatment complexity within this cohort. These results should be interpreted as risk factors specific to this female population, rather than as gender-specific predictors, thereby offering a basis for more individualized decision-making for this subgroup, which is increasingly recognized as having unique perioperative management considerations.

### High-risk factors: stone location and unique female anatomical and physiological characteristics

Stone location (renal vs. ureteral) was theoretically the strongest risk factor in this study (OR = 6.67). This significant difference profoundly reflects the unique anatomical and physiological characteristics of the female upper urinary tract: ① Susceptibility to Elevated Renal Pressure and Poor Drainage: The renal pelvis volume in females is relatively small, and the anatomical structure of the collecting system is more enclosed. When stones are located in the kidney (especially in calyceal diverticula), they are more likely to cause urinary drainage obstruction and increased intrarenal pressure, inducing pyelovenous and lymphatic backflow, which promotes the entry of bacteria and endotoxins into the bloodstream, significantly increasing the risk of systemic infection [9, 10]. The risk associated with renal-ureteral stones was secondary (OR = 3.38), reflecting the transitional risk characteristics of proximal obstruction, combining both intrarenal hypertension and incomplete obstruction. ② High Incidence of Infection Stones and Hormonal Influences: The prevalence of infection stones in females reaches 17.22% (compared to only 8.27% in males) [3, 6]. Although estrogen in premenopausal women may provide some protection by inhibiting calcium oxalate crystal formation (a view supported by ex vivo studies such as that by Peerapen et al. [7]), the sharp decline in estrogen levels after menopause weakens the urothelial defense function, reduces urinary citrate excretion, and removes a key inhibitory factor, thereby significantly increasing the risk of renal stone formation. This conclusion is consistent with the research by Prochaska et al. [11]. ③ Physiological Changes During Pregnancy: Physiologic hydroureter during pregnancy leading to urinary stasis is an important predisposing factor for stone formation and persistent infection in women of reproductive age, indirectly increasing the risk of stone formation [12]. While the anatomical and physiological characteristics described above provide a background for the increased susceptibility in female patients, it is important to emphasize that female sex serves primarily as a risk modifier rather than an independent determinant of treatment complexity. The absolute risk in individual patients is strongly influenced by more proximate factors, including preoperative urine culture status, the presence of infection stones, and operative parameters such as surgical duration and intrarenal pressure control. This distinction has clinical relevance, as it suggests that the potential for risk reduction lies in optimizing modifiable factors such as preoperative antibiotic therapy, staged procedures when indicated, and careful intraoperative pressure management rather than viewing female sex as an unchangeable risk factor.

### Risk factors: the central role of bacterial/fungal infection

Bacterial/fungal infection represents another critical risk factor (OR = 3.30, 95% CI: 1.42–7.62). Its underlying mechanism is highly consistent with the pathological mechanism of urinary calculi complications: ① Calculi serve as a substrate for bacterial biofilm colonization: Bacterial biofilms formed on the surface of calculi can evade antibiotic clearance, leading to persistent infection and a systemic inflammatory response [13, 14]. ② Endotoxemia and sepsis risk: Endotoxins released by Gram-negative bacteria (e.g., Escherichia coli, a common pathogen in female urinary tract infections [UTIs]) are key factors triggering systemic inflammatory response syndrome (SIRS) and sepsis [15]. Some studies [5, 16] have indicated that female sex is a risk factor for sepsis following stone surgery. The shorter urethral anatomy in females predisposes them to UTIs. Notably, infection with urease-producing bacteria (e.g., Proteus species) not only causes urine alkalization but also directly promotes the formation and accumulation of infectious stones, such as struvite (magnesium ammonium phosphate) stones [6], thereby establishing a vicious cycle of “infection-stone-reinfection”. In this study, the infection rate in the complex course group was significantly higher at 34.58% (compared to only 10.71% in the control group), strongly supporting the close association between infectious stones and the risk of sepsis [17].

### Intermediate risk factor: the value of infection biomarkers (hs-CRP, PCT) in early warning of sepsis in female patients

Elevated hs-CRP and PCT, serving as profound biomarkers for infection and inflammatory response, were identified as intermediate risk factors, holding particular significance in the early warning of sepsis risk in female patients: ① High Specificity of PCT for Gram-Negative Bacterial Infection: PCT is a sensitive marker in response to endotoxins from Gram-negative bacteria, and its elevation is closely associated with the risk of bacteremia and sepsis. The PCT cutoff value established in this study (≥ 0.65 ng/mL, approximating the 75th percentile in the complex course group) approached the commonly used diagnostic threshold for sepsis (> 0.5 ng/mL) [18]. It should be noted that this cutoff value was derived from the distribution of biomarker levels within the current cohort rather than from an externally validated threshold. Similarly, the hs-CRP cutoff (≥ 7.3 mg/L) was based on the median value in the complex course group. These data-driven cutoffs serve as exploratory reference points for hypothesis generation and should be interpreted with caution when applied to other clinical settings. Studies have confirmed that procalcitonin has good diagnostic performance for urosepsis, with a sensitivity of up to 87% [18], which aligns closely with the susceptibility of female patients to infections caused by Gram-negative bacteria such as Escherichia coli, which often form biofilms [14]. ② As a non-specific but sensitive acute-phase reactant, persistently elevated hs-CRP indicates a significant systemic inflammatory state and the extent of tissue damage. During the progression of urosepsis, elevated hs-CRP levels are often directly associated with organ dysfunction [19]. The independent predictive value of both PCT and hs-CRP further supports, from the perspective of the inflammatory response, the strong relevance of infection as a driving factor in the occurrence of complex clinical courses in female patients undergoing surgery for upper urinary tract stones. However, external validation in independent cohorts is required before these cutoffs can be applied for risk stratification in broader clinical practice.

### Consideration of negative findings: the weakening effect of technological advancements on traditional risk factors

Age, stone area, ureteral stenosis, BMI, metabolic syndrome (diabetes, hypertension, fatty liver), and acute renal insufficiency were not identified as independent risk factors in the current theoretical risk framework, although they still warrant attention as potential confounding factors. The potential reasons are analyzed as follows: ① Confounding Effects and the Presence of Stronger Predictors: For instance, univariate analysis indicated that the complex course group was older (57.3 vs. 53.1 years), but its odds ratio (OR 1.024, *P* = 0.104) in multivariate analysis was not statistically significant. This may be because advanced age is often associated with an increased risk of infection (e.g., due to declined immunity) and a higher proportion of renal stones [20], effects which were likely masked by the core high-risk factors (infection, stone location) identified in this study. The varying relationships between age and infectious stones reported in different studies [6] might stem from population selection bias or age-related changes in stone composition. ② Measurement Limitations and Confounding Control: Potential subjective errors may exist in the imaging-based measurement of stone area. Although ureteral stenosis theoretically increases surgical difficulty and complication risk (*P* = 0.242 in this study), its lack of independent effect might be attributed to sample size limitations, interference from confounding factors, or effective counteraction by advancements in endourological technology.③Effective Counteraction by Advancements in Endourological Technology: Modern minimally invasive urological techniques (e.g., slim/small-caliber flexible/rigid ureteroscopes, personalized selection of ureteral access sheaths with various calibers, intraoperative ultrasound/fluorescence navigation) have significantly enhanced the ability to manage complex anatomy (e.g., ureteral stenosis, tortuous ureters in obese patients) and achieve precise lithotripsy, effectively reducing the direct impact of these factors on surgical treatment outcomes.④ Modern Management and Secondary Nature of Metabolic Factors: Although elevated BMI is associated with stone formation (e.g., by promoting uric acid stones through insulin resistance [21]) and increased surgical difficulty (median 24.20 in the adverse outcome group vs. 24.00 in the control group), it did not translate into a significant prognostic risk (*P* = 0.519), suggesting that modern technology can overcome the technical challenges posed by obesity. The lack of significance for metabolic comorbidities such as diabetes, hypertension, and fatty liver may be related, on one hand, to improvements in modern disease management [22]. On the other hand, it might suggest that their mechanisms of action in postoperative complications among female stone patients differ from those in males. For example, Xiao Y et al. [23] found that female diabetic patients received greater protection from ACE inhibitors against kidney stone disease compared to male diabetic patients. The incidence of acute renal insufficiency in the complex course group of this study was 14.02%, one-third of whom (5/15 cases) had concurrent sepsis. Its P-value of 0.081 in multivariate analysis supports the notion that it is mostly a secondary consequence of severe infection (sepsis) or long-term obstruction, rather than a primary driving factor, consistent with the view of Chien TM et al. [24].

### Clinical significance and application of the preoperative risk stratification framework

If this risk stratification framework is validated in future studies, it may serve as a basis for preoperative assessment. For patients identified as high risk (i.e., those with renal stones and active infection), clinicians may consider adopting more proactive intervention strategies, such as extending the duration of preoperative targeted antibiotic therapy, planning staged procedures, or selecting surgical equipment that effectively controls intrarenal pressure [25, 26]. For intermediate-risk patients (e.g., those with elevated inflammatory markers), enhanced perioperative monitoring and inflammation management may be warranted. This risk-stratified management approach, pending external validation, has the potential to optimize medical resource allocation and support individualized patient care.

### limitations

This study has several limitations. First, the single-center retrospective design may introduce selection bias. Second, the risk stratification framework has not undergone external validation, and its generalizability requires confirmation through prospective multicenter studies. Third, stone composition was not analyzed, and potential confounding factors such as surgeon experience were not included. Fourth, the follow-up period was relatively short, precluding assessment of long-term outcomes.

Regarding model construction, although multicollinearity diagnostics (VIF < 3) revealed no significant collinearity, the inclusion of seven independent variables with a sample size of 219 may affect model stability. The effect estimates therefore require confirmation in larger cohorts or through external validation.

The use of a composite outcome, while helpful for comprehensive assessment of treatment complexity, carries certain limitations. Its three components, namely preoperative stent placement, frequent readmissions, and prolonged hospital stay, reflect treatment decision-making, resource utilization, and recovery trajectory, respectively. However, they differ in pathophysiological mechanisms and clinical severity, and aggregating them into a single endpoint may obscure heterogeneity. Importantly, infection-related variables (bacterial/fungal infection, hs-CRP, PCT) are directly linked to the “preoperative stent placement” component, which may lead to overestimation of their predictive strength. Therefore, their independent predictive role should be interpreted with caution. Nevertheless, the composite outcome still facilitates identification of preoperative risk factors consistently associated with a complicated course, supporting its utility as a risk stratification tool. Future studies may analyze each component separately or apply weighting according to clinical relevance to further refine prediction.

Furthermore, as this study included only female patients without direct comparison with males, whether the identified risk factors differ by sex remains unclear. Sex-related differences in anatomy, hormonal status, and microbiological susceptibility may modulate these factors, warranting comparative studies including both sexes.

## Conclusion

Bacterial/fungal infection, stone location (renal stones), elevated hs-CRP, and elevated PCT are effective preoperative predictors of treatment complexity in female patients with upper urinary tract stones. The proposed exploratory risk stratification framework may aid in the identification of high-risk patients and offers preliminary evidence to support individualized surgical strategies and perioperative management plans, though prospective validation is required before clinical implementation.

## Data Availability

The datasets used or analyzed during the current study are available from the corresponding author on reasonable request.

## Abbreviations

hs-CRP: high-sensitivity C-reactive protein
PCT: procalcitonin
BMI: body mass index
PCNL: Percutaneous Nephrolithotomy
RIRS: Retrograde Intrarenal Surgery
WBC: white blood cell
ORs: odds ratios
95% CIs: 95% confidence intervals
VIF: variance inflation factor
